# Lignosulfonic Acid Sodium Is a Noncompetitive Inhibitor of Human Factor XIa

**DOI:** 10.3390/ph14090886

**Published:** 2021-08-31

**Authors:** Srabani Kar, Page Bankston, Daniel K. Afosah, Rami A. Al-Horani

**Affiliations:** 1 Division of Basic Pharmaceutical Sciences, College of Pharmacy, Xavier University of Louisiana, New Orleans, LA 70125, USA; skar@xula.edu (S.K.); pclemons@xula.edu (P.B.); 2 Department of Chemistry and Biochemistry, Washington and Lee University, Lexington, VA 24450, USA; dafosah@wlu.edu

**Keywords:** factor XIa, allosteric inhibitor, anticoagulant, lignin, sulfonate

## Abstract

The anticoagulant activity of lignosulfonic acid sodium (LSAS), a non-saccharide heparin mimetic, was investigated in this study. LSAS is a relatively safe industrial byproduct with similar polyanionic characteristics to that of heparin. Human plasma clotting assays, fibrin polymerization testing, and enzyme inhibition assays were exploited to investigate the anticoagulant activity of LSAS. In normal human plasma, LSAS selectively doubled the activated partial thromboplastin time (APTT) at ~308 µg/mL. Equally, LSAS doubled APTT at ~275 µg/mL in antithrombin-deficient plasma. Yet, LSAS doubled APTT at a higher concentration of 429 µg/mL using factor XI-deficient plasma. LSAS did not affect FXIIIa-mediated fibrin polymerization at 1000 µg/mL. Enzyme assays revealed that LSAS inhibits factor XIa (FXIa) with an IC_50_ value of ~8 μg/mL. LSAS did not inhibit thrombin, factor IXa, factor Xa, factor XIIIa, chymotrypsin, or trypsin at the highest concentrations tested and demonstrated significant selectivity against factor XIIa and plasmin. In Michaelis–Menten kinetics, LSAS decreased the V_MAX_ of FXIa hydrolysis of a tripeptide chromogenic substrate without significantly changing its K_M_ indicating an allosteric inhibition mechanism. The inhibitor also disrupted the generation of FXIa–antithrombin complex, inhibited factor XIIa-mediated and thrombin-mediated activation of the zymogen factor XI to FXIa, and competed with heparin for binding to FXIa. Its action appears to be reversed by protamine sulfate. Structure–activity relationship studies demonstrated the advantageous selectivity and allosteric behavior of LSAS over the acetylated and desulfonated derivatives of LSAS. LSAS is a sulfonated heparin mimetic that demonstrates significant anticoagulant activity in human plasma. Overall, it appears that LSAS is a potent, selective, and allosteric inhibitor of FXIa with significant anticoagulant activity in human plasma. Altogether, this study introduces LSAS as a promising lead for further development as an anticoagulant.

## 1. Introduction

**Figure 1:**
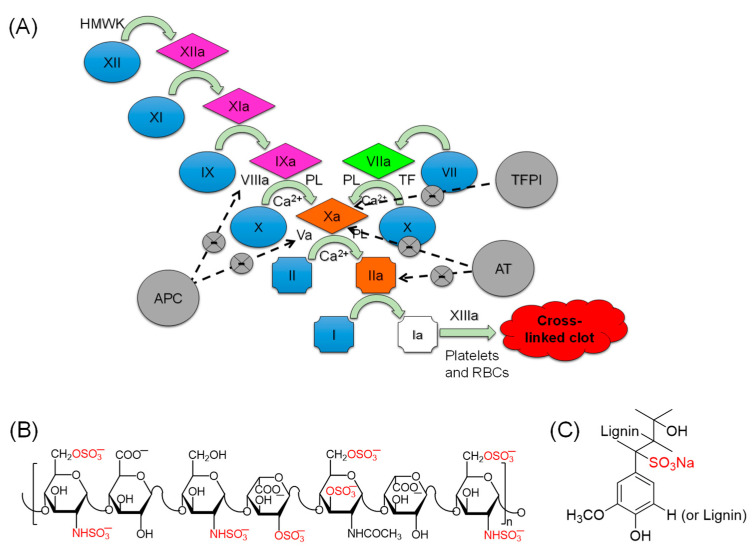
(**A**) A waterfall representation of the coagulation cascade in which the serine proteases of the intrinsic pathway (FXIIa, FXIa, and FIXa), the extrinsic pathway (FVIIa), and the common pathway (thrombin and FXa) work in a cascade fashion to form a clot that subsequently gets rigidified by FXIIIa-mediated fibrin cross linking. (**B**) A representative chemical structure of heparins, a mixture of linear polysaccharides that are largely decorated with negative charge (sulfate and carboxylate). The sulfate groups represent the fundamental structural requirements needed for heparins to act as anticoagulants via activating endogenous AT. (**C**) A representative structure of lignosulfonic acid sodium (LSAS) polymer that is proposed in this study as a platform to develop alternatives to heparins for use as clinically relevant and safe anticoagulants by targeting proteins in the intrinsic pathway of coagulation. High molecular weight kininogen (HMWK), phospholipid (PL), tissue factor pathway inhibitor (TFPT), antithrombin (AT), activated protein C (APC), red blood cells (RBCs).

The coagulation process is a series of biochemical transformations, all of which are mainly catalyzed by serine proteases including factors IIa (FIIa), VIIa (FVIIa), IXa (FIXa), Xa (FXa), XIa (FXIa), and XIIa (FXIIa). Factor XIIIa (FXIIIa), however, is a transglutaminase. The coagulation process is triggered by the intrinsic pathway or the extrinsic pathway (Figure 1A) and is highly regulated by serpins, serine protease inhibitors. Examples of these endogenous serpins are antithrombin (AT; inhibitor of FXa and thrombin), tissue factor pathway inhibitor (TFPI; inhibitor of FXa), and activated protein C (APC; inhibitor of factors V and VIIIa), among others. Dysregulation of such process may result in either thrombosis or hemophilia. In particular, thrombotic diseases include venous thrombosis such as pulmonary embolism and deep vein thrombosis, or arterial thrombosis such as ischemic heart attacks and stroke [1]. Their treatment/prevention entails the use of anticoagulants which includes heparins. Unfractionated heparins are mixtures of highly sulfated polysaccharides that have been used as anticoagulants for more than 90 years [2]. Heparins promote such activity primarily by activating AT. Heparins are approved for prophylaxis and for treatment of pulmonary embolism and venous thromboembolism and thromboembolic complications related to atrial fibrillation. They are also used as anticoagulants for blood transfusions and extracorporeal circulation. Interestingly, porcine heparins are the only anticoagulants approved in the US for open heart surgeries and kidney dialysis [2,3].

Given the heterogeneity of heparin mixtures (Figure 1B), at least 81 deaths and 785 injuries were linked to contaminated heparins in 2008 in the US [4]. Currently, there is a serious concern over the supply of heparins because of the deadly African swine fever outbreak, which has affected the Chinese pipeline since August 2018 and has led to the death of ~2 million pigs [5]. This is in addition to the other side effects of heparins such as the life-threatening potential of excessive bleeding, heparin-induced thrombocytopenia, osteoporosis, and the intra- and inter-patient response variations [6]. Thus, alternatives to heparin therapy are urgently needed.

In this study, we have identified LSAS polymer (Figure 1C) as an allosteric inhibitor of human FXIa, as revealed by the corresponding in vitro experiments of chromogenic tripeptide substrate hydrolysis assays as well as Michaelis–Menten kinetics. LSAS inhibited human FXIa with an IC_50_ value of ~8 μg/mL and a maximal efficacy of ~100%. The molecule has demonstrated substantial selectivity toward FXIa over other coagulation, digestive, and fibrinolytic proteases. The inhibitor also selectively prolonged the APTT of human plasma. Interestingly, LSAS inhibited the physiological function of FXIa, i.e., FIX activation, and inhibited FXIIa-mediated and thrombin-mediated activation of FXI in a concentration-dependent manner. The inhibitor interfered with the formation of FXIa–antithrombin complex in the presence of heparins, indicating that it may compete with heparins for binding to or in the vicinity of the anion-binding site(s) of FXIa. Similarly, it was also demonstrated that the molecule may engage with heparin-binding domain(s) on FXIa in competition studies with heparins. The action of LSAS was found to be reversed by protamine sulfate, the same clinical antidote for heparins. The inhibition and mechanistic behaviors of LSAS are unique compared with its non-sulfonated or acetylated derivatives. Overall, this study introduces LSAS as a promising lead for further development as an anticoagulant.

## 2. Results and Discussion

### 2.1. Effects of LSAS on Clotting Times of Normal and Deficient Human Plasmas 

Plasma clotting assays including APTT, PT, and TT are commonly used to study the anticoagulation potential of new procoagulant enzyme inhibitors under in vitro settings. The APTT evaluates the effect of potential anticoagulants on the contact/intrinsic pathway-mediated clotting which involves factors IXa, XIa, and XIIa. The PT evaluates the effect of potential anticoagulant on FVIIa in the extrinsic pathway of coagulation. The TT measures the effect of potential anticoagulant on thrombin-induced plasma clotting.

**Figure 2:**
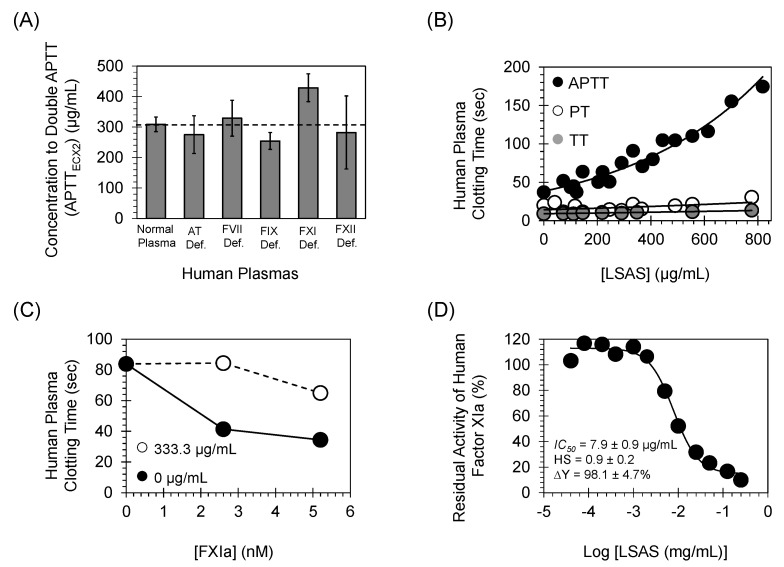
(**A**) The effect of LSAS on the APTT of normal human plasma and human plasmas deficient of antithrombin (AT), FVII, FIX, FXI, or FXII. (**B**) The effects of LSAS on the APTT (intrinsic pathway), PT (extrinsic pathway), and TT (thrombin-induced clotting) of normal human plasma. (**C**) The APTT profile of FXI-deficient human plasma following the addition of 2.6 nM or 5.2 nM, in the presence (○) or absence (●) of LSAS. (**D**) The inhibition profile of human FXIa by LSAS, as spectrophotometrically measured in the chromogenic tripeptide substrate hydrolysis assay.

Initially, we evaluated LSAS effect on APTT using normal human plasma, as well as human plasmas deficient of AT, FVII, FIX, FXI, and FXII. In these assays, the effect of different concentrations of LSAS on APTT of normal human plasma and deficient human plasmas (Figure 2A) and (Table 1) were measured, as described in earlier studies [7,8]. Results indicated that LSAS concentration-dependently doubled APTT of normal plasma at 308.8 ± 23.9 μg/mL. Using human plasma deficient of antithrombin, FVII, FIX, and FXII, LSAS concentration-dependently doubled the APTT at similar concentrations of 275.0 ± 62.0, 329.1 ± 58.7, 254.4 ± 27.5, and 282.2 ± 119.8 μg/mL, respectively. Nevertheless, a higher concentration of LSAS was needed to double the APTT of human plasma deficient of FXI which was about 428.9 ± 45.6 μg/mL, an increase of about 1.5-fold. Furthermore, it was found that ~980 μg/mL and >>500 μg/mL of LSAS were required to double the PT and TT of human plasma (Figure 2B) and (Table 1). These results suggest that LSAS is likely to promote its anticoagulant properties by targeting the intrinsic pathway of coagulation, particularly by targeting human FXIa. This was further confirmed by comparing the plasma effect profile of LSAS with those of approved and experimental anticoagulants (Table S1). Under our conditions, unfractionated heparins (UFH) were found to affect both APTT as well as PT at concentrations of 0.68 μg/mL and 2.53 μg/mL, respectively, while 10.1 μg/mL was needed from UFH to double APTT in human plasma deficient of antithrombin confirming that antithrombin is the clinical target of UFH. In contrast, there was no statistically significant difference between LSAS concentrations needed to double the APTT of normal plasma or that of AT-deficient plasma (308.8 ± 23.89 μg/mL versus 275.0 ± 62.0 μg/mL), suggesting that antithrombin is not a target for LSAS. Similarly, argtroban (thrombin inhibitor) and rivaroxaban (FXa inhibitor) both affect APTT and PT at similar concentrations (Table S1); however, LSAS was found to primarily affect APTT (index of selectivity was >3-fold). In fact, the LSAS plasma profile appears to be similar to that of anti-F11 (FXIa inhibitor) and/or C6B7 (FXIIa inhibitor) (Table S1). However, using FXII-deficient plasma, the concentration of LSAS to double the APTT was similar to that using normal human plasma (308.8 ± 23.89 μg/mL versus 282.2 ± 119.8 μg/mL). The above results suggest that human FXIa is the more likely target for LSAS. 

To confirm that FXIa is involved in mediating the anticoagulant effect of LSAS, we studied the effect of adding LSAS (333.3 μg/mL) on FXIa-induced clotting of FXI-deficient human plasma. Figure 2C shows that FXI-deficient human plasma clotted at 84 s, however, adding 2.6 nM or 5.2 nM of human FXIa accelerated the clotting to occur at 41.4 s or 34.4 s, respectively. Nevertheless, the addition of 333.3 μg/mL of LSAS significantly postponed the FXIa-induced clotting of FXI-deficient human plasma by 2-fold when clotting was provoked by 2.6 nM FXIa or by 1.9-fold when clotting was induced by 5.2 nM FXIa. Overall, these results further establish that LSAS anticoagulant activity in human plasma is due to its effect on the intrinsic coagulation pathway, primarily by inhibiting FXIa.

### 2.2. Inhibition of Human FXIa by LSAS in Chromogenic Substrate Assay

LSAS was investigated for its potential to inhibit FXIa hydrolysis of the chromogenic tripeptide substrate S-2366 under the physiological conditions of 37 °C and pH 7.4, as reported earlier [7,8,9]. In this assay, the polymer was found to inhibit FXIa in a dose-dependent fashion (Figure 2D), and the inhibition of FXIa was fitted using the logistic Equation (1) resulting in an IC_50_ value of 7.9 ± 0.9 μg/mL (0.15 ± 0.02 μM) and an efficacy of 98.1 ± 4.7% (Table 2). Previous reports indicated that UFH inhibits human FXIa with an IC_50_ value of 3.15 ± 0.75 μg/mL (0.21 ± 0.05 μM) and an efficacy value of only 30% [10]. Thus, LSAS and UFH appear to have a comparable IC_50_ toward inhibiting human FXIa, yet LSAS is a more effective inhibitor.

### 2.3. Selectivity Studies: Inhibition Potential of LSAS toward Other Coagulation Proteins 

**Figure 3:**
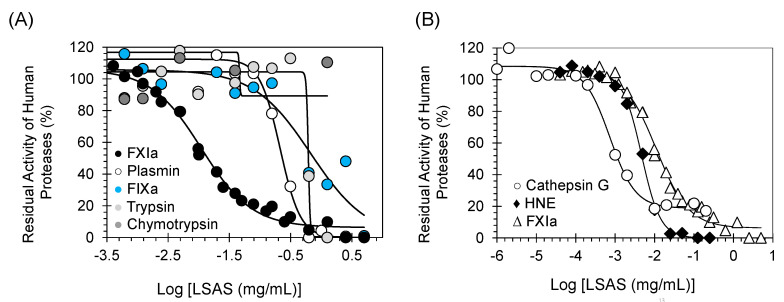
(**A**) Direct inhibition of serine proteases (including human FXIa) by LSAS. (**B**) Direct inhibitions of FXIa and the inflammatory serine proteases cathepsin G and HNE by LSAS. The inhibition of FXIa, plasmin, FIXa, trypsin, chymotrypsin, HNE, and cathepsin G by LSAS was studied as described in Methods and Materials. Solid lines are the sigmoidal dose–response fits (Equation (1)) to the data to obtain the inhibition parameters.

To determine the selectivity profile of LSAS, we also evaluated its inhibitory potential toward other coagulation proteins including thrombin, FXa, FIXa, and FXIIa using the corresponding chromogenic tripeptide substrate hydrolysis tests under near physiologic conditions, as reported in our previous studies [7,8,9,11]. In these tests, the inhibition potential was spectrophotometrically measured from residual enzyme activity in the presence of different concentrations of LSAS (Figure 3A). Moreover, LSAS activity against human FXIIIa was also investigated using the bi-substrate, fluorescence-based transglutamination assay, as described earlier [12]. Based on the highest concentration tested of LSAS against the above enzymes, the calculated IC_50_ values are estimated to be >125 μg/mL for thrombin, >125 μg/mL for FXa, >673 μg/mL for FIXa, and >12.5 μg/mL for FXIIIa (Table 2). The polymer was found to inhibit FXIIa with an IC_50_ value of 714.0 ± 107.5 suggesting a selectivity index of at least 90-fold. Overall, the above results further confirm that LSAS is a selective inhibitor of human FXIa, as determined in the above in vitro assays.

### 2.4. Selectivity Studies: Inhibition Potential of LSAS toward Other Serine Protease Important for Fibrinolysis, Digestion, and Inflammation 

We also evaluated LSAS inhibitory potential against other proteases including plasmin, trypsin, chymotrypsin, human neutrophil elastase (HNE), and cathepsin G using the corresponding chromogenic substrate hydrolysis assays under near physiologic conditions, as reported in our earlier studies [7,8,9,11,12,13,14,15]. In these assays, the inhibition potential was determined by spectrophotometric measurement of the residual protease activity in the presence of varying concentrations of LSAS (Figure 3A,B). Based on the highest concentrations of LSAS tested against the above enzymes, the calculated IC_50_ values are estimated to be 212.5 ± 25.8 μg/mL for plasmin, >600 μg/mL for trypsin, and >2000 μg/mL for chymotrypsin (Table 2). Interestingly, the polymer was also found to inhibit HNE with an IC_50_ value of 4.7 ± 0.2 μg/mL as well as cathepsin G with an IC_50_ value of 0.73 ± 0.11 μg/mL. Thus, the above results indicate that LSAS is a selective inhibitor for human FXIa over fibrinolytic, coagulation, and digestive enzymes, but may exert anti-inflammatory effects by inhibiting HNE and cathepsin G. A dual-acting anticoagulant/anti-inflammatory agent is a novel therapeutic concept given the crosstalk between coagulation and inflammation.

### 2.5. Reversibility of FXIa Inhibition by LSAS. Protamine as a Reversal Agent

**Figure 4:**
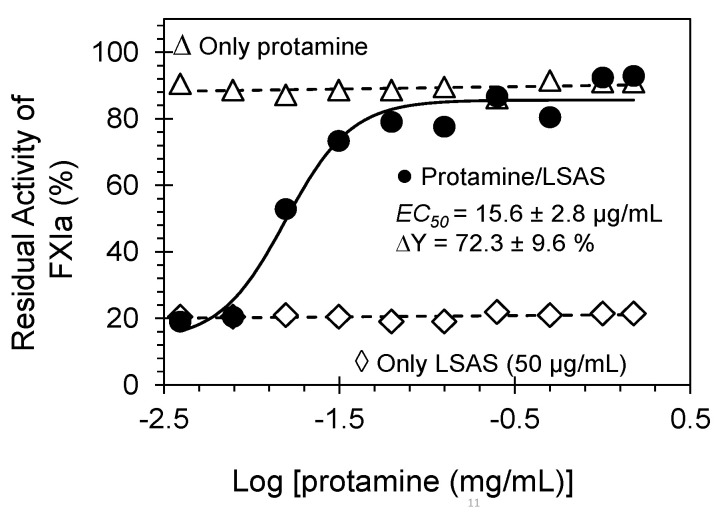
Reversibility of LSAS inhibition of FXIa by protamine sulfate. Shown is the restored FXIa activity (%), which was inhibited by 50 μg/mL of LSAS, in the presence of ascending concentration of protamine (●). Shown also are the effects of only protamine sulfate (0.0039 mg/mL–1.5 mg/mL) on the activity of FXIa (Δ) and the effect of only LSAS (50 μg/mL) on FXIa. The restored FXIa activity profiles were spectrophotometrically established at and 37 °C and pH 7.4. Solid lines represent fits by the dose–response Equation (2) to get the *EC_50_*.

A key feature for developing new anticoagulants is the ability to reverse anticoagulant activity. To evaluate whether FXIa inhibition by LSAS can be reversed, we studied this phenomenon using protamine. Protamine is a clinically used Arg-rich polypeptide that reverses the anticoagulant activity of UFH and low molecular weight heparins (LMWHs) [14]. FXIa was initially incubated with a saturating concentration of LSAS (50 μg/mL) and the recovery of FXIa activity by the potential reversal agent was spectrophotometrically studied by the hydrolysis of FXIa chromogenic tripeptide substrate at 37 °C and pH 7.4 (Figure 4). As a result, the inhibitory effect of LSAS was effectively reversed by ~70% with an EC_50_ value of 15.6 ± 2.8 μg/mL. Thus, LSAS inhibition of FXIa is reversible which helps in further developing this polymer to a more clinically relevant molecule. 

### 2.6. Effect of LSAS on the Physiological Function of FXIa, i.e., Activation of FIX 

**Figure 5:**
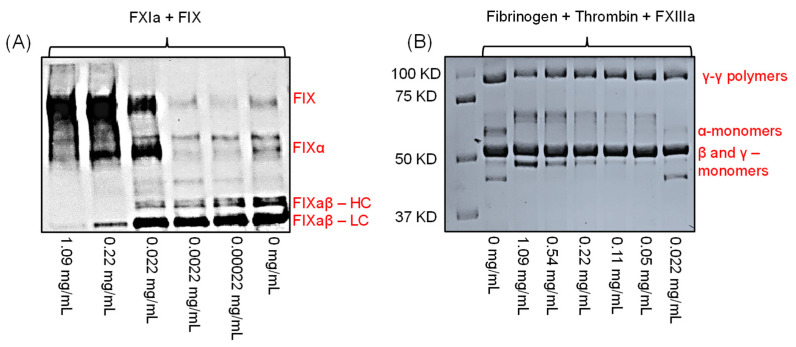
(**A**) Effect of LSAS on FXIa-mediated activation of FIX using Western blotting under reducing conditions. LSAS concentrations used were 0, 0.00022, 0.0022, 0.022, 0.22, and 1.09 mg/mL. Shown is activation of FIX to FIXα then to FIXaβ by cleaving the peptide bonds, Arg145–Ala146 and Arg180–Val181, respectively. FIXaβ appears as two bands: one band for the heavy chain (FIXaβ–HC) and the other band for the light chain (FIXaβ–LC), under reducing conditions. (**B**) Effect of LSAS on FXIIIa-mediated fibrin crosslinking as evaluated by SDS-PAGE under reducing conditions. Results indicate that the above concentrations did not affect the γ–γ polymer formation, and thus, the lack of effect on FXIIIa or even thrombin.

Although LSAS inhibited the hydrolysis of the tripeptide substrate S-2366 by FXIa, we wanted to establish its physiological relevance by studying its effect on FIX, the physiological substrate of FXIa. During the coagulation process, FXIa activates FIX (Figure 1) by sequentially cleaving two bonds, Arg145–Ala146 and Arg180–Val181, so as to generate FIXaβ [16]. In the presence of calcium ions and phospholipids, FIXaβ and factor VIIIa subsequently form the intrinsic tenase complex to activate factor X to FXa, which, at the end, amplifies the generation of thrombin [17]. To confirm the physiological relevance of LSAS inhibitory activity toward FXIa, we studied FXIa activation of FIX in the absence and presence of LSAS using Western blotting (Figure 5A). The figure shows that LSAS dose-dependently (0–1.09 mg/mL) inhibited the formation of FIXα (an intermediate FIX) and the fully activated FIX, i.e., FIXaβ (heavy chain (FIXaβ-HC) and light chain (FIXaβ-LC) as revealed by the absence of the corresponding bands. The above results indicate that LSAS’s inhibition of FXIa is physiologically relevant and it significantly occurs at a concentration as low as 22 μg/mL, which is very close to the IC_50_ determined in the chromogenic substrate hydrolysis assay.

### 2.7. Effect of LSAS on FXIIIa-Mediated Polymerization of Fibrin(ogen)

To confirm the lack of LSAS effect on FXIIIa’s physiological function, we monitored FXIIIa-mediated fibrin cross-linking following activation of fibrinogen by thrombin using gel electrophoresis (Figure 5B). This experiment revealed that LSAS inhibited the formation of γ–γ polymers (~117 kD) at the highest concentration tested of 1.09 mg/mL. Accordingly, this result further confirms the results obtained above in the bisubstrate, fluorescence-based trans-glutamination assay.

### 2.8. Effect of LSAS on FXIa Interaction with Macromolecules

**Figure 6:**
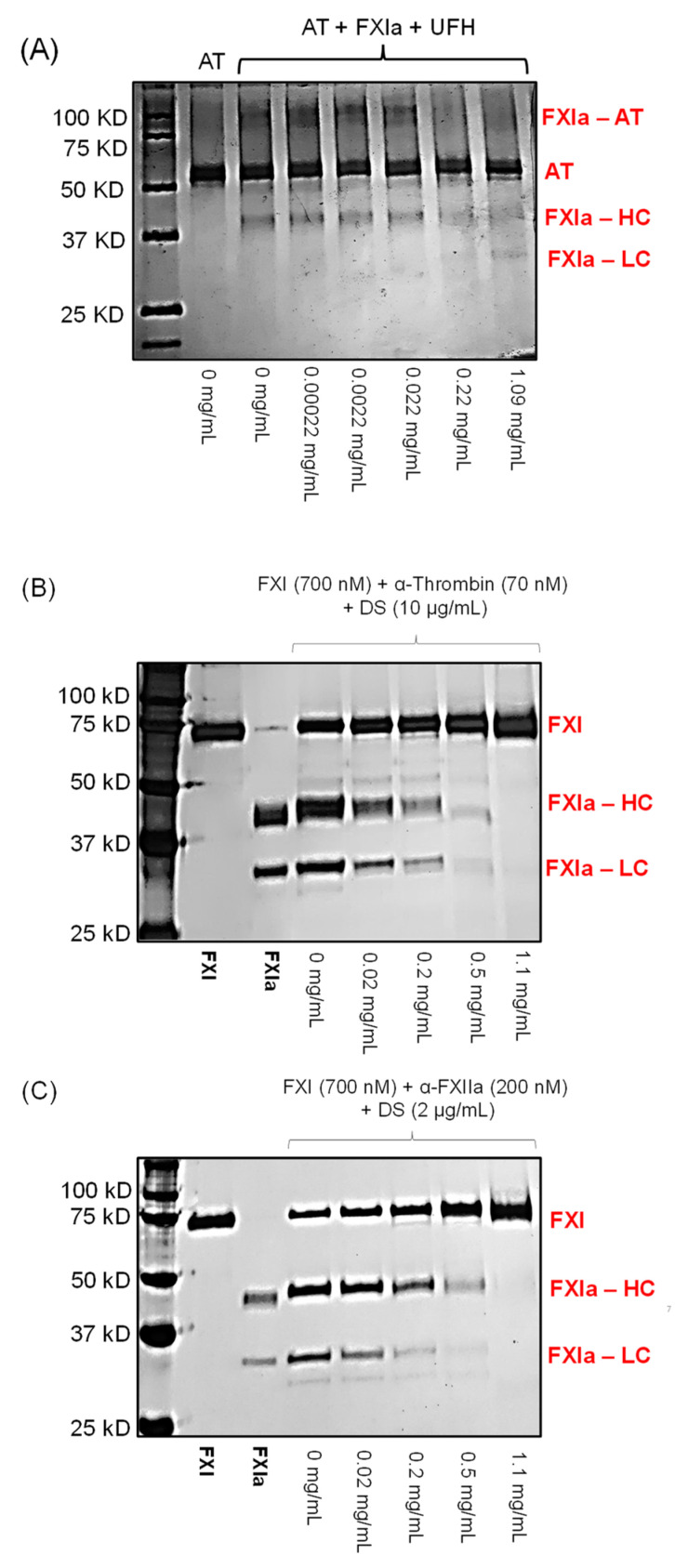
Effect of different concentrations of LSAS (0–1.1 mg/mL) on FXI(a) interactions with macromolecules including the formation of FXIa–AT complex in the presence of UFH (**A**), activation of FXI to FXIa by thrombin (**B**), and activation of FXI to FXIa by FXIIa (**C**). Concentrations of LSAS used were 0, 0.00022, 0.0022, 0.022, 0.22, and 1.1 mg/mL. LSAS disrupted all interactions suggesting that it recognizes both the zymogen FXI and its enzyme FXIa, possibly by binding to an anion-binding site on FXI and similar site on the catalytic domain of FXIa.

To investigate the effect of LSAS beyond the inhibition of the catalytic activity of FXIa, we studied three intermolecular interactions in which FXI(a) interacts with another partner protein. First, it is well known that FXIa can be inhibited by AT in a reaction that is accelerated by UFH via a bridging- or template-based mechanism [18]. In this process, a denaturation-resistant complex is formed between the reactive center loop of AT and the active site of FXIa. In this complex, the light chain of FXIa acylates AT by forming a covalent bond. Figure 6A depicts SDS-PAGE showing a 90 kDa band which indicates the formation of the FXIa-LC–AT complex (lane 3) in the absence of LSAS. However, the presence of ascending concentrations of LSAS (0–1.09 mg/mL) inhibited the formation of the same complex. Indeed, the formation of the complex appears to be substantially decreased at the highest concentration tested of 1.09 mg/mL. At concentrations 0.22 mg/mL and 1.09 mg/mL, the band of 90 kDa complex on SDS-PAGE significantly diminished and that of FXIa-LC reappeared at the 30 kDa mark (lanes 7 and 8). These results indicate that LSAS interferes with FXIa-AT complex formation, likely by competing with UFH for its anion binding sites on apple 3 domain or on the catalytic domain.

Furthermore, it is well reported that the zymogen FXI is activated to the enzyme FXIa by the actions of FXIIa or thrombin in processes that are accelerated by negatively charged surfaces such as inorganic polyphosphates or dextran sulfate [19,20]. In particular, dextran sulfate is a sulfated polysaccharide that accelerates FXI activation via a template mechanism in which the dextran sulfate binds to the anion binding sites on both the activators, i.e., FXIIa or thrombin and the substrate, i.e., FXI [20,21,22,23,24,25]. Figure 6B shows that LSAS concentration-dependently inhibited the thrombin-mediated activation of FXI, as demonstrated by the diminished intensity of FXIa-LC and FXIa-HC bands (lanes 5–8), over the concentration range of 0.02–1.1 mg/mL. Likewise, Figure 6C shows that LSAS concentration-dependently inhibited the FXIIa-mediated activation of FXI, as demonstrated by the decreased intensity of the bands of the heavy and light chains of FXIa (lanes 5–8), over the concentration range of 0.02–1.1 mg/mL. Taken together, LSAS has been shown to inhibit the dextran sulfate-facilitated activation of FXI to FXIa by FXIIa and thrombin at similar concentrations to those identified in the preceding experiments. This suggests that LSAS likely competes with the dextran sulfate for the anion binding sites on apple 3 domain or on the catalytic domain of FXIa. Thus, the above results indicate that LSAS potentially binds to an allosteric site on FXI(a) rather than the active site. Similar to SPGG and SCI, two sulfated non-saccharide mimetics of heparins [7,15], the binding site is potentially the anion-binding site in the catalytic domain.

### 2.9. Competition Studies with UFH

**Figure 7:**
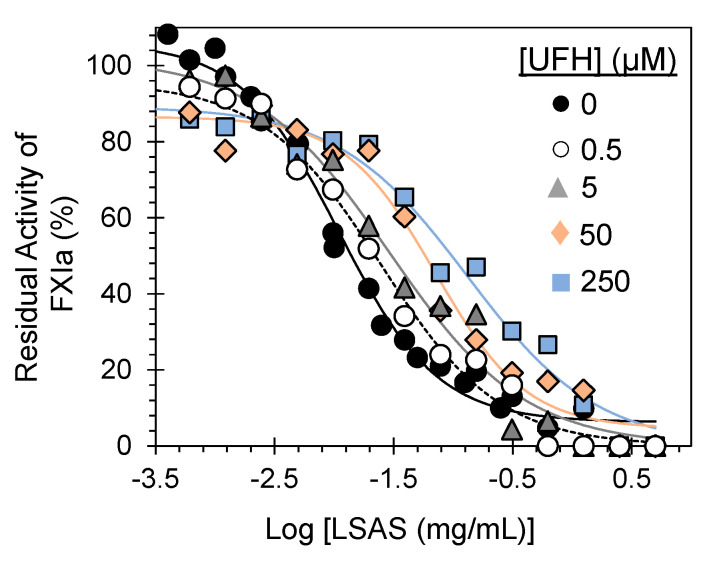
Competitive inhibition of human FXIa by LSAS in the presence of UFH. The FXIa inhibition was measured spectrophotometrically at 37 °C and pH 7.4. Solid lines represent fits by the dose–response Equation (1) to calculate the inhibition parameters. The concentrations of UFH elected for the experiment were 0 (●), 0.5 (○), 5 (▲), 50 (♦), and 250 (■) μM.

UFH binds to FXIa in two sites: the apple 3 domain (Lys252, Lys253, and Lys255) and the catalytic domain (Lys529, Arg530, Arg532, Lys535, and Lys539) [26]. To identify whether LSAS engages with the heparin-binding sites (anion-binding sites) on FXIa, the IC_50_s of FXIa inhibition by LSAS were determined using the FXIa chromogenic substrate hydrolysis assay used in Section 2.2 (Figure 7 and Table 3), in the presence of ascending UFH concentrations (0–250 μM). The rationale here is that FXIa inhibition by LSAS should be progressively diminished as the UFH concentration increases, if the two anionic polymers (UFH and LSAS) compete well. Figure 7 depicts the change in dose–response profiles of FXIa inhibition by LSAS in the presence of UFH at 37 °C and pH 7.4. As the concentration of UFH ascended from 0 to 250 μM, the IC_50_ of FXIa inhibition by LSAS increased from 11.0 ± 1.8 μg/mL to 131.6 ± 22.7 μg/mL, which is equivalent to about a 12-fold decrease in the LSAS potency with no significant change in the inhibition efficacy. This suggested a substantial competition between LSAS and UFH. Overall, the results indicate that LSAS competes with UFH for its binding sites on FXIa, which is consistent with the results of the previous experiments. An important point to mention here is that, from the AT-deficient plasma studies, we believe that the plasma concentration of AT may not affect the LSAS behavior. The LSAS APTT_ECX2_ in normal plasma was 308.8 ± 23.9 µg/mL, which is similar to the APTT_ECX2_ of 275.0 ± 62.0 µg/mL for LSAS in AT-deficient study (Table 1). Nevertheless, the effect of plasma AT concentration on LSAS action in vivo remains to be tested.

### 2.10. Mechanism of FXIa Inhibition by LSAS: Michaelis–Menten Kinetics

**Figure 8:**
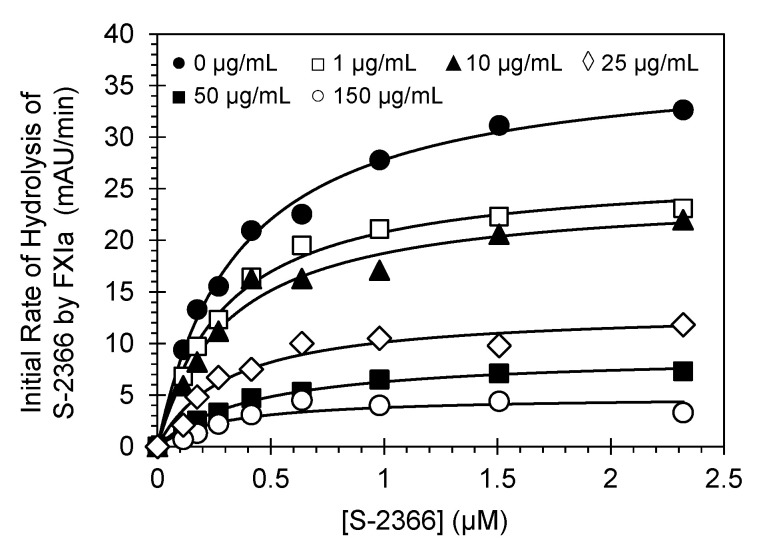
Michaelis–Menten kinetics of the S-2366 chromogenic tripeptide hydrolysis by human FXIa in the presence of different concentrations of LSAS. The initial rate of hydrolysis at different substrate concentrations was measured in pH 7.4 buffer using the wild type of full length FXIa. The concentrations of LSAS selected in this experiment were 0 (●), 1 (□), 10 (▲), 25 (◊), 50 (■), and 250 μg/mL (○). Lines represent nonlinear regressional fits to the data by the Michaelis–Menten Equation (3).

To understand the mechanistic basis of LSAS inhibition of FXIa, Michaelis–Menten kinetics of S-2366 hydrolysis by FXIa was performed in the presence of LSAS at pH 7.4 and 37 °C, as reported earlier [7,15]. Figure 8 shows the initial rate profiles in the presence of LSAS (0–150 μg/mL). Each profile shows a rectangular hyperbolic dependence. The profiles were fitted using Equation (3) to give the apparent K_M_ and V_MAX_ (Table 4). The results suggest that K_M_ for S-2366 remained essentially unchanged in the presence or absence of LSAS, while the V_MAX_ decreased steadily from 37.7 ± 1.0 mAU/min in the absence of LSAS to 4.9 ± 0.7 mAU/min at 150 μg/mL of LSAS (~7.7-fold decrease). Therefore, LSAS brings about spatial changes in the active site of FXIa, which induces a substantial dysfunction in the catalytic domain without affecting the formation of the Michaelis complex. This indicates that LSAS is an allosteric inhibitor of FXIa.

### 2.11. Effects of Acetylation and Desulfonation on the Activity of LSAS towards FXIa

**Figure 9:**
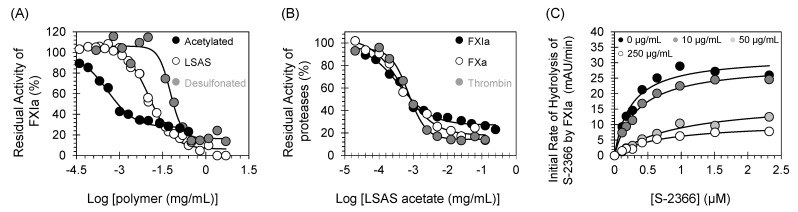
(**A**) Direct inhibition of FXIa by LSAS and its acetylated and desulfonated derivatives. The inhibition of FXIa by acetylated LSAS (●), LSAS (○), and desulfonated LSAS (●) was studied at 37 °C and pH 7.4. Solid lines represent sigmoidal dose–response fits using Equation (1) to the data to calculate the inhibition parameters. (**B**) Direct inhibition of coagulation serine proteases (thrombin (●), FXa (○), and FXIa (●)) by acetylated LSAS. (**C**) Michaelis–Menten kinetics of S-2366 chromogenic tripeptide hydrolysis by FXIa in the presence of desulfonated LSAS. The initial rate of hydrolysis at different substrate concentrations was determined using wild-type full-length FXIa, in pH 7.4 buffer. The concentrations of desulfonated LSAS chosen in the study were 0 (●), 10 (●), 50 (●), and 250 μg/mL (○). Solid lines represent nonlinear regression fits to the data by the Michaelis–Menten Equation (3).

To understand the structural features that contribute to LSAS behavior, we studied two closely related polymers: acetylated lignosulfonic acid and desulfonated lignin. We evaluated their inhibitory effect toward FXIa using the chromogenic substrate hydrolysis assay under physiological conditions at which LSAS inhibitory potency was evaluated against FXIa. Figure 9A reveals that the acetylated as well as the desulfonated lignin inhibited FXIa, yet the acetylated form was more potent than LSAS with an IC_50_ value of 0.39 ± 0.1 μg/mL (20-fold more potent), whereas the desulfonated form was less potent than LSAS with an IC_50_ value of 53.9 ± 16.3 μg/mL (6.8-fold less potent) (Figure 9A, Table 5). Interestingly, despite the high potency of the acetylated form of LSAS, it was not selective as it potently inhibited both thrombin and factor Xa with IC_50_ values of 0.73 ± 0.04 and 0.48 ± 0.09, respectively (Figure 9B). Furthermore, the desulfonated form of LSAS appears to behave differently as it increased the K_M_ and decreased V_MAX_, which perhaps indicates it is a mixed inhibitor (Figure 9C) and (Table 6). Although this phenomenon requires further investigation, the desulfonated form is nevertheless about 7-fold less potent than LSAS, which emphasizes the significance of the sulfonate group for potency as well as the allosteric mechanism of inhibition.

## 3. Materials and Methods

### 3.1. Materials 

LSAS (Mw~52,000), LSAS acetate, and lignin (low sulfonate content ≤ 3.6%) were purchased from Sigma Aldrich (St. Louis, MO, USA). For the enzyme kinetics studies, human plasma clotting enzymes, fibrinolytic enzymes, and digestive enzymes were purchased from Haematologic Technologies (Essex Junction, VT, USA). HNE was purchased from Elastin Products Company (Owensville, MO, USA). Cathepsin G was obtained from Enzo Life Sciences (Farmingdale, NY, USA). The chromogenic substrates for thrombin, FXa, FIXa, FXIIa, and plasmin were purchased from Biomedica Diagnostics (Windsor, NS, Canada). Trypsin chromogenic substrate (S-2222) and FXIa chromogenic substrate (S-2366) were purchased from Diapharma (West Chester, OH, USA). The chromogenic substrates S-1384 for HNE and S-7388 for cathepsin G were from Sigma Aldrich. Dithiothreitol (DTT), *N,N*-dimethylcasein, and dansyl-cadaverine were also from Sigma Aldrich (St. Louis, MO, USA). UFH and protamine sulfate were from Milipore-Sigma, whereas dextran sulfate (MW ca > 500,000) and Coomassie Brilliant Blue for gel electrophoresis were from Fisher Scientific ((Waltham, MA, USA)). AntiF11 (mouse monoclonal antibody from Abnova^TM^) for plasma studies was also from Fisher Scientific. Antithrombin and fibrinogen were from Haematologic Technologies (Essex Junction, VT, USA). FXIIaα and antibodies for Western blot were purchased from Enzyme Research Laboratories (South Bend, IN, USA).

Stock solutions of thrombin, FXIa, FXIIa, plasmin, chymotrypsin, and trypsin were all prepared in 50 mM TrisHCl buffer, pH 7.4, containing 0.02% Tween80, 0.1% PEG8000, and 150 mM NaCl. Stock solution of FIXa was prepared in 20 mM TrisHCl buffer, pH 7.4, containing 0.02% Tween80, 0.1% PEG8000, 2.5 mM CaCl_2,_ 100 mM NaCl, and 33% *v*/*v* ethyleneglycol. Stock solution of FXa was prepared in 20 mM TrisHCl buffer, pH 7.4, containing 100 mM NaCl, 2.5 mM CaCl_2_, 0.1% PEG8000, and 0.02% Tween80. Stock solution of FXIIIa was prepared in 50 mM Tris-HCl buffer containing 0.02% Tween80, 0.1% PEG8000, 1 mM CaCl_2_, 100 mM NaCl, and 2 mg/mL *N,N*–dimethylcasein. Stock solution of HNE was prepared by reconstituting 1 mg with 100 µL of 1:1 200 mM Na acetate:glycerol buffer of pH 5, which was then diluted using HEPES buffer, pH 7.4, containing 125 mM HEPES, 100 mM NaCl, and 0.125% Triton-X 100.

For the clotting time (APTT, PT, and PT) testing, deficient and pooled normal human plasma were obtained from George King Bio-Medica (Overland Park, KS, USA). APTT reagent containing ellagic acid, PT reagent containing thromboplastin-D, and 0.025 M solution of CaCl_2_ were obtained from Thermo Fisher Scientific (Waltham, MA, USA). Experiments in this manuscript were reproduced at least two times. 

### 3.2. Effect of LSAS on Clotting Times of Deficient and Normal Human Plasmas

Plasma clotting assays of activated partial thromboplastin time (APTT), prothrombin time (PT), and thrombin time (TT) are commonly used to study the anticoagulant activity of enzyme inhibitors. APTT measures the effect of the new potential therapeutic entity on the contact/intrinsic pathway-driven clotting which involves FXIIa, FXIa, and FIXa. PT measures the effect of the new potential therapeutic entity on the extrinsic pathway of coagulation which involves FVIIa, whereas TT measures the effect of potential anticoagulant on thrombin-induced clotting. These experiments were conducted using the BBL Fibrosystem fibrometer (Becton−Dickinson, Sparles, MD, USA), as documented in earlier studies [7,8]. For the APTT assay, 90 μL of citrated human plasma was mixed with 10 µL of LSAS (or the vehicle) and 100 μL of prewarmed 0.2% ellagic acid. After incubation for 4 min at 37 °C, clotting was provoked by adding 100 μL of prewarmed 0.025 M CaCl_2_, and the time to clotting was noted. For the PT assay, thromboplastin-D was prepared by adding 4 mL of distilled water, and then, the resulting mixture was warmed to 37 °C. A 90 µL volume of citrated human plasma was then mixed with 10 μL of LSAS (or the vehicle), and was subsequently incubated for 30 s at 37 °C. Following the addition of 200 μL of prewarmed thromboplastin-D reagent, the time to clotting was noted. For the TT assay, 10 µL of LSAS was brought up to 200 µL with citrated human plasma, incubated for 3 min at 37 °C followed by the addition of 100 µL of thrombin time reagent, and the time to clotting was recorded. In the three assays, about 9 or more concentrations of LSAS were used to construct concentration vs. effect profiles. The data were plotted to a quadratic trendline, which was used to estimate the LSAS concentration of LSAS needed to double the clotting time. Clotting times using 10 µL of highly purified water (negative control) were also measured in a similar manner. To determine the effect on the APTT of deficient human plasmas, the APTT assay was repeated, using plasmas deficient of AT, factor IX (FIX), factor VII (VII), factor XII (FXII), or factor XI (FXI). The positive control used in APTT and PT assays were (1) UFH, antithrombin activator; (2) argatroban, a clinically used thrombin inhibitor; (3) rivaroxaban, a clinically used FXa inhibitor, (4) anti-F11, an experimental FXIa inhibitor; and (5) C6B7, an experimental FXIIa inhibitor. To confirm the FXIa-dependent effect of LSAS in human plasma, the APTT assay was performed using FXI-deficient human plasma to which human FXIa (2.6 nM or 5.2 nM) was added in the presence and the absence of LSAS (333.3 µg/mL).

### 3.3. Inhibition of Human FXIa in Chromogenic Tripeptide Substrate Hydrolysis Assay by LSAS

Direct inhibition of human FXIa was investigated by the corresponding chromogenic tripeptide substrate hydrolysis testing, as described in previous reports [8,9], at 37 °C and pH 7.4. Each well of the 96-well microplate had 85 μL of the buffer to which 5 μL of human FXIa (final concentration in the well is 0.765 nM) and 5 µL of LSAS were added. Following 10-min incubation, 5 μL of FXIa substrate (final concentration in the well is 345 μM) was added and the residual FXIa activity was obtained from the initial rate of increase in A_405_ nm. Stock of LSAS was serially diluted to obtain a concentration range of (0–5 mg/mL) in the wells. Relative residual activity of human FXIa at each LSAS concentration was calculated from the ratio of FXIa activity in the presence and absence of LSAS. Logistic Equation (1) was used to plot the dose dependence of residual human FXIa activity so as to obtain the efficacy (ΔY%; y-axis) and the potency (IC_50_; x-axis) of the inhibition.

(1) $$Y\;={\;Y}_{0}+\frac{Y_{M}-Y_{0}}{1+10^{\left(\log{\left[I\right]}_{0}-{\log\;\mathrm{IC}}_{50}\right)\left(\mathrm{HS}\right)}}$$

In this equation, Y is the ratio of residual FXIa activity in the presence of LSAS to that in its absence, Y_0_ and Y_M_ are the minimum and maximum values of the fractional residual FXIa activity, respectively, IC_50_ is the concentration of LSAS that leads to 50% inhibition of FXIa activity, and HS is the Hill slope. Y_0_, Y_M_, IC_50_, and HS measurements are determined by nonlinear curve fitting of the data.

### 3.4. Effect of LSAS on Other Enzymes in the Coagulation Process

The inhibition potential of LSAS against FIXa, FXa, FXIIa, and thrombin was also investigated using the corresponding chromogenic tripeptide substrate hydrolysis assays reported in earlier papers [7,11]. Briefly, to each well of a 96-well microplate containing 185 μL of 20–50 mM Tris-HCl buffer, pH 7.4, containing 0.02% Tween80, 0.1% PEG8000, and 100–150 mM NaCl, and at either 37 °C (FIXa, FXa, and FXIIa) or 25 °C (thrombin), 5 μL of LSAS (0–5.0 mg/mL in the well) (or the vehicle) and 5 μL of the enzyme were added. The final concentrations of the enzymes were 89 nM (FIXa), 1.09 nM (FXa), 5 nM (FXIIa), and 6 nM (thrombin). Following 10-min incubation, 5 μL of FIXa substrate (final conc. is 850 µM), FXa substrate (125 µM), FXIIa substrate (125 µM), or thrombin substrate (50 µM) was added, and the residual enzyme activity was determined from the initial rate of increase in A_405_ nm. Relative residual enzyme activity as a function of LSAS concentration was calculated, if significant inhibition was observed. Similarly, to determine the effect of LSAS on human FXIIIa, a bi-substrate, fluorescence-based trans-glutamination assay was performed as we documented in earlier studies [12]. Generally, 1 μL of LSAS was diluted with 87 μL of pH 7.4 buffer (50 mM Tris-HCl, 1mM CaCl_2_, 100 mM NaCl, and 2 mg/mL *N,N–*dimethylcasein), and 5 μL DTT (20 mM) at 37 °C followed by the addition of 2 μL of human FXIIIa (0.3 μM) and incubation for 10 min. After the addition of 5 μL of dansylcadaverine (2 mM), the activity of FXIIIa was monitored by measuring the initial rate of increase in fluorescence emission (λ_Ex._ = 360 nm and λ_Em._ = 490 nm). The inhibition parameters were calculated using Equation (1), in case 50% or more of the enzyme was inhibited by LSAS.

### 3.5. Effect of LSAS on Fibrinolysis, Digestive, and Inflammatory Serine Proteases

The inhibition potential of LSAS against plasmin, trypsin, chymotrypsin, and human neutrophil elastase (HNE) was also studied using the corresponding chromogenic tripeptide substrate hydrolysis assays as documented in our previous reports [7,8,9,11,12,13]. Briefly, to each well of a 96-well microplate containing 185 μL (trypsin, chymotrypsin, and HNE) or 85 µL (plasmin) of 20–50 mM Tris-HCl buffer, pH 7.4, containing, 0.02% Tween80, 0.1% PEG8000, and 100–150 mM NaCl at 37 °C was added 5 μL of LSAS (0–5.0 mg/mL in the well) (or the vehicle) and 5 μL of the enzyme. The final concentrations of the enzymes were 20 nM (plasmin and HNE), 30 nM (cathepsin G), and 0.5 µg/mL (trypsin and chymotrypsin). Following 5-min incubation, 5 μL of Spectrozyme PL (final conc. 50 nM), S-2222 (trypsin substrate; 0.65 mM), *N*-succinyl Ala-Ala-Pro-Phe-p-nitroanilide (chymotrypsin substrate; 0.91 mM), S7388 (HNE; 2 mM), or S-7388 (cathepsin G; 2 mM) was added and the residual enzyme activity was measured from the initial rate of increase in A_405_ nm. The inhibition parameters were calculated using Equation (1), in case 50% or more of the enzyme was inhibited by LSAS.

### 3.6. Reversibility of FXIa Inhibition by LSAS

To evaluate the in vitro reversibility of LSAS’s inhibitory effect, the restored FXIa activity profiles were constructed using an ascending protamine sulfate concentration [14] in the presence of LSAS (50 μg/mL), which inhibited FXIa ~100%. The measurements were performed by S-2366 hydrolysis assay using a microplate reader (Fisherbrand™ accuSkan™ FC Filter-Based Microplate Photometer) at physiological conditions of 37 °C and pH 7.4. Generally, each well of the 96-well microplate had 80 μL of 50 mM Tris-HCl buffer of pH 7.4 containing 0.02% Tweeen80, 0.1% PEG8000, and 150 mM NaCl to which 5 μL of LSAS (or the reference), 5 μL of FXIa (0.765 nM), and 5 μL of protamine sulfate (1 mg/mL–0.0078 mg/mL) were successively added. After a 10 min incubation, 5 μL S-2366 substrate (345 μM) was added and the restored FXIa activity was measured from the initial rate of increase in A_405_ nm. Relative restored FXIa activity at each concentration of protamine sulfate was calculated from the ratio of FXIa activity in the absence and presence of protamine sulfate. Logistic Equation (2) was used to fit the concentration dependence of restored FXIa activity to obtain the efficacy (ΔY%; y-axis) of the reversing process and the effective concentration of protamine sulfate required to restore 50% of FXIa activity at specific inhibitor concentration (EC_50;_ x-axis).

(2) $$Y\;={\;Y}_{0}+\frac{Y_{M}-Y_{0}}{1+10^{\left(\log{\left[R\right]}_{0}-{\log\;\mathrm{EC}}_{50}\right)\left(\mathrm{HS}\right)}}$$

### 3.7. Effect of LSAS on the Physiological Function of Human FXIa, i.e., FIX Activation

A Western blot experiment was done as reported earlier [7,15]. Plasma FIX (200 nM) was incubated with human FXIa (40 nM) at room temperature, in the presence of different concentration of LSAS (0.0002, 0.002, 0.02, 0.2, 1.09 mg/mL) in 50 mM HEPES buffer supplemented with 5 mM CaCl_2_. After 30 min incubation, SDS-PAGE loading buffer having DTT was added, fractionated on 10% polyacrylamide SDS gels, and subsequently transferred to nitrocellulose membrane. The primary and secondary antibodies used were goat anti-human FIX polyclonal IgG and horseradish peroxidase-conjugated anti-goat IgG, respectively. Detection was by chemiluminescence. The relative positions of protein or protein part bands were determined using Western blot of known standards. 

### 3.8. Effect of LSAS on FXIIIa-Mediated Polymerization of Fibrin(ogen)

The effect of LSAS polymer on FXIIIa-mediated fibrin polymerization was further studied by SDS-PAGE technique, as documented previously [7,12]. A solution containing 1.75 mg/mL fibrinogen and 0.9 µg/mL FXIIIa in 50mM Tris HCl buffer of pH 7.4 containing 10 mM CaCl_2_ was incubated with different concentration of LSAS (0.0002, 0.002, 0.02. 0.2, 0.5, and 1.1 mg/mL), and then clotted in the presence of human α-thrombin (1.25 µg/mL). At room temperature, the clots were incubated for 24 h before adding the denaturing buffer of 25 mM NaH_2_PO4, 1.9% (*w*/*v*) SDS, 5.7 M urea, and 1.9% (*w*/*v*) DTT, and then incubated overnight at room temperature. Samples were boiled in a water bath for 10 min, centrifuged at 12,000 *g* at 20 °C for 3 min, and then the supernatants were examined by SDS-PAGE on homogeneous 10 % cross-linked gels. For visualization, the gels were stained with Coomassie Brilliant Blue.

### 3.9. Effect of LSAS Polymer on FXIa–AT Complex Formation

The effect of LSAS polymer on the FXIa–AT complex formation was performed in HEPES buffer enriched with 5 mM of CaCl_2_, as documented previously [7,27]. Briefly, FXIa (300 nM) was pre-incubated with different LSAS concentrations (0.0002, 0.002, 0.02, 0.2, 1.09 mg/mL) at room temperature for 5 minutes, and then combined with human AT (2 µM) in the presence of heparin (2 µM). The resulting mixture was incubated for an additional 30 minutes. Then, samples were quenched using SDS-PAGE loading gel buffer containing DTT and subjected to electrophoresis on 10 % SDS-PAGE. Visualization was by silver stain. 

### 3.10. Effect of LSAS Polymer on Thrombin-Mediated Activation of FXI

Thrombin activation of the zymogen FXI to the enzyme FXIa was studied by SDS-PAGE experiment, as documented previously [7,21,22,23,24,28]. FXI (700 nM) was incubated with dextran sulfate (10 µg/mL) and with α-thrombin (70 nM) in the presence of different LSAS concentrations (0.02. 0.2, 0.5, 1.1, 2 mg/mL) in HEPES buffer enriched with 5 mM of CaCl_2_. Following 60 mins incubation, the reaction at each LSAS concentration was quenched using polybrene (6 µg/mL) and argatroban (2 µM). Samples were then put into the reducing sample buffer, size fractionated on 10% SDS-PAGE gel, and lastly visualized by silver staining. 

### 3.11. Effect of LSAS Polymer on FXI Activation by FXIIa-Mediated Activation of FXI

FXIIa activation of the zymogen FXI to the enzyme FXIa was analyzed by SDS-PAGE experiment, as documented previously [7,21,22,23,24,28]. FXI (700 nM) was incubated with dextran sulfate (10 µg/mL) and with α-FXIIa (200 nM) in the presence of different LSAS concentrations (0.02. 0.2, 05, 1.1 and 2 mg/mL) in HEPES buffer enriched with 5 mM of CaCl_2_. Following 60 mins incubation, the reaction at each LSAS concentration was quenched using polybrene (6 µg/mL) and corn trypsin inhibitor (1 µM). Samples were then put into the reducing sample buffer, size fractionated on 10% SDS-PAGE gel, and lastly visualized by silver staining. 

### 3.12. Competition Studies of LSAS with UFH

Inhibition of FXIa by LSAS was performed in the presence of UFH using the 96–well platform. A 5 µL solution of LSAS (0–5 mg/mL) and 5 µL of human FXIa (0.765 nM) with 5µL of UFH (0, 0.5, 5, 50, 150, and 250 µM) in 80 µL of 50 mM TrisHCl buffer, pH 7.4, containing 0.02% Tween80, 0.1% PEG8000, and 150 mM NaCl, and was then incubated for 5 mins at 37 °C. Following incubation, 5 µL of FXIa substrate (final concentration in the well is 345 µM) was added and the initial change in A_405_ nm was determined. The concentration dependence of the fractional residual FXIa activity at each concentration of UFH was fitted by Equation (1) to calculate the apparent concentration of LSAS required to reduce FXIa activity to 50% of its initial value in the presence of UFH.

### 3.13. Michaelis–Menten Kinetics for S-2366 Hydrolysis by Human FXIa in the Presence of LSAS

The initial rate of S-2366 hydrolysis by human FXIa was obtained from the linear increase in A_405_ nm relevant to the consumption of less than 10% of the chromogenic substrate, as documented previously [7]. The initial rate was measured as a function of different concentrations of S-2366 (2.3−0.11 mM) in the presence of a fixed concentration of LSAS in 20 mM Tris−HCl buffer, pH 7.4, containing 0.02% Tween80, 0.1% PEG8000, and 150 mM NaCl at 37 °C. The experiment was conducted at five concentrations of LSAS (0, 1, 10, 25, 50 and 150 µg/mL). The data were fitted using the standard Michaelis–Menten Equation (2) to determine the V_MAX_ (the maximum hydrolysis reaction velocity; y-axis) and the K_M_ (the affinity of the substrate to the active site of FXIa; x-axis).

(3) $$V=\frac{V_{\mathrm{MAX}}\left[S\right]\;}{K_{M}+\left[S\right]}$$

### 3.14. Effects of Acetylation and Desulfonation on the Activity of LSAS towards FXIa

The inhibitory potential of a corresponding acetylated or desulfonated derivatives of LSAS toward human FXIa was evaluated as described above for LSAS. FXIa activity in the absence and presence of acetylated LSAS or desulfonated LSAS was measured from the initial rate of increase in A_405_ nm. Stocks were serially diluted to obtain a concentration range of (0–5 mg/mL) in the wells. Relative residual FXIa activity at each concentration of the inhibitor was calculated from the ratio of FXIa activity in the presence and absence of the potential inhibitor. Logistic Equation (1) was used to fit the concentration dependence of residual FXIa activity so as to get the efficacy (ΔY%; y-axis) and the potency (IC_50_; x-axis) of inhibition.

## 4. Conclusions and Future Directions

In this study, we identified LSAS, an industrial lignin-based byproduct, as a polymeric allosteric inhibitor of human FXIa. FXIa is the current popular target to design new generations of anticoagulants that are not associated with a bleeding risk because it plays a more active role in the pathology of thrombosis and not in the physiology of hemostasis [1,7,10,15,27]. LSAS demonstrates considerable potency and selectivity for FXIa over other clotting enzymes namely, thrombin, FIXa, FXa, FXIIa, and FXIIIa, as well as other related serine proteases of trypsin, chymotrypsin, and plasmin. Its action can also be reversed by protamine, the same clinical arginine-rich antidote used to reverse the action of heparins. 

A mechanistic phenomenon that adds to LSAS’s clinical relevancy is its allosteric inhibition behavior. Allostery serves an exceptional prospect of highly selective recognition, which physiology generally recruits to an advantage [29]. Allosteric sites tend to be less conserved than orthosteric sites in a group of homologous proteins. For example, allosteric sites of thrombin, FIXa, FXa, and FXIa display considerable sequence variability [30,31], although they possess a similar trypsin-like, catalytic site specificity. This considerably facilitates selective targeting of an allosteric site, and therefore, minimizes the potential of off-target side effects. In this vein, Michaelis–Menten kinetics revealed that LSAS possesses a classical allosteric inhibition mechanism (Figure 8). LSAS allostery arises from binding to the anion-binding site(s) on FXIa as shown by the competitive binding studies in the presence of UFH (Figure 7) and the disruption of FXIa–AT complex in the presence of dextran sulfate (Figure 6A). Interestingly, the molecule inhibits the catalytic activity of FXIa not only as evaluated by using a small chromogenic tripeptide (Figure 2D), but also as evaluated using the zymogen FIX (Figure 5A), which further establishes its physiological relevance. LSAS also inhibits the activation of the zymogen FXI to its active form by thrombin and FXIIa (Figure 6B,C), which takes place in the presence of anionic molecules such as heparins, polyphosphate, and dextran sulfate [20,21,22,23,24,25] further supporting its projected binding site(s). Importantly, LSAS demonstrates significant selective anticoagulant activity in human plasma by prolonging only the APTT (which entails FXIa) and not the PT or TT (Figure 2A,B, Table 1). This once again establishes its selective inhibition profile. 

Together, LSAS offers several advantages as an anticoagulant: (1) it is readily available which makes it commercially similar to heparins from an economic point of view; (2) it comes with a reduced potential of deadly contamination with other super-sulfated polysaccharides and eliminates the issue of heparins’ supply shortage in the US, as happened recently; and (3) it is associated with a little-to-no risk of bleeding in comparison to heparins because of its primary target (FXIa) and unique mechanism (allostery), which makes its use for the same clinical indications of heparins even safer. LSAS also only causes acute toxicity at a very large oral dose (LD_50_ = 6,030 mg/kg in mice) [32]. In fact, given the need for safer anticoagulants that do not cause bleeding complications to be used in high-risk patients such as those with chronic kidney diseases or atrial fibrillation [33,34,35,36,37], some future studies will focus on designing small molecules based on the structure of LSAS to study their structure–activity relationship so as to put forward a more clinically relevant allosteric, sulfonated, small molecule inhibitors of human FXIa as anticoagulants that are not associated with bleeding consequences. Future studies will also include establishing the FXIa-mediated anticoagulant effect of LSAS in human blood using thromboelastography. They will also include testing LSAS’s anticoagulant activity in appropriate models of arterial and venous thrombosis animal models as well as tail bleeding assays. 

A recent publication reported that drugs targeting FXIa as a member of the contact activation enzymes may serve as a potential treatment for COVID-19 patients [38]. Earlier, inhibition of contact activation (including FXIa), in nonhuman primates, prevented death from *Staphylococcus aureus*-induced systemic inflammatory response syndrome [39]. Furthermore, targeting FXI(a)/FXII(a) axis by therapeutic tools has been demonstrated to prevent systemic inflammation, coagulopathy, and mortality in experimental sepsis [40,41]. Interestingly, we found in this study that LSAS inhibits HNE, an inflammatory serine protease the overactivity of which contributes to a host of cardiopulmonary diseases [13], with an IC_50_ value of 4.7 ± 0.2 µg/mL (Table 2). Furthermore, we also found that LSAS inhibits another inflammatory protease, i.e., cathepsin G [42] with an IC_50_ value of 0.73 ± 0.11 µg/mL (Table 2). In addition to their role in inflammation, both HNE and cathepsin G have been found to have procoagulant effects. Both proteases have been shown to activate coagulation factors V and X. [43,44] Additionally, cathepsin G has been found to contribute to coagulation by activating factor VIII to its active form FVIIIa, promoting thrombin generation and fibrin formation, as well as via the activation of platelets. [45].

Together, these studies suggest that polymeric molecules or small molecules derived from LSAS polymer can be pharmaceutically designed and developed as adjunct treatment for COVID-19 and other infectious pandemics or epidemics that are associated with inflammation and coagulopathy.

Lastly, because LSAS is a large polymer, and therefore, its inhibition potential can be confused with its aggregation tendency, we attempted to measure its minimum aggregation concentration in FXIa inhibition studies buffer (which contains the aggregation-preventing surface-active agent Tween80). We measured the potential of aggregation using increasing concentrations of LSAS at different UV wavelengths (340, 405, 450, 540, and 620 nm), as previously reported [46], and found that its FXIa IC_50_ and its human plasma APTT × 2 take place at a much lower concentration than that at which aggregation may take place (Figure S1). This establishes the viability of LSAS to undergo further chemical and pharmacological studies as an anticoagulant or as a platform to design more clinically relevant and safer anticoagulants as well as to design dual-acting agents against pathologies associated with excessive coagulopathy and inflammation.

## Figures and Tables

**Table 1:** The effect of LSAS on the clotting times (APTT, PT, and TT) of different human plasmas ^a^.

Type of Human Plasma	(LSAS) (µg/mL) to Double Clotting Time
**APTT assay**	**APTT_EC×2_**
Normal	308.8 ^b^ ± 23.9 ^c^
Deficient of AT	275.0 ± 62.0
Deficient of FVII	329.1 ± 58.7
Deficient of FIX	254.4 ± 27.5
Deficient of FXI	428.9 ± 45.6
Deficient of FXII	282.2 ± 119.8
**PT assay**	**PT_EC×2_**
Normal	980.1 ± 145.0
**TT assay**	**TT_EC×2_**
Normal	>>500

^a^ Prolongation of clotting times as a function of LSAS concentration in three clotting assays; ^b^ The effective concentrations of LSAS needed to double the clotting times when coagulation triggered by the intrinsic pathway (APTT_EC×2_), the extrinsic pathway (PT_EC×2_), and thrombin (TT_EC×2_). In each experiment, the concentration is the average of three measurements. ^c^ Errors represent ± 1 S.E.

**Table 2:** Inhibition of clotting factors by LSAS ^a^.

Enzyme	IC_50_ (µg/mL)	HS	∆Y (%)
**Thrombin**	>125	*ND ^c^*	*ND*
**FXa**	>125	*ND*	*ND*
**FIXa**	> 673	*ND*	*ND*
**FXIa**	7.9 ± 0.9 ^b^	0.9 ± 0.2	98.1 ± 4.7
**FXIIa**	714.0 ± 107.5	2.4 ± 0.5	92.6 ± 4.5
**FXIIIa**	>12.5	*ND*	*ND*
**Plasmin**	212.5 ± 25.8	2.6 ± 0.8	112.4 ± 6.1
**Trypsin**	>600	*ND*	*ND*
**Chymotrypsin**	>2000	*ND*	*ND*
**HNE**	4.7 ± 0.2	1.8 ± 0.2	104.9 ± 2.1
**Cathepsin G**	0.73 ± 0.11	1.3 ± 0.3	89.7 ± 4.0

^a^ The inhibition parameters were obtained following non-linear regression analysis of human enzymes direct inhibition, at 37 °C, in Tris-HCl buffers of pH 7.4–8.0. Inhibition was determined by measuring the residual enzyme activity (UV or fluorescence). ^b^ Errors represent ± 1 S.E. ^c^ Not determined.

**Table 3:** Inhibition of human FXIa by LSAS in the presence of UFH under physiologically relevant conditions ^a^.

UFH (µM)	IC_50_ (µg/mL)	HS	∆Y (%)
**0**	11.0 ± 1.8 ^b^	1.1 ± 0.2	99.8 ± 5.4
**0.5**	24.6 ± 3.7	0.9 ± 0.1	95.6 ± 4.5
**5**	28.9 ± 5.6	0.8 ± 0.1	102.1 ± 5.2
**50**	70.6 ± 11.6	1.1 ± 0.2	82.0 ± 4.5
**150**	91.8 ± 21.3	0.7 ± 0.1	90.6 ± 5.3
**250**	131.6 ± 22.7	0.8 ± 0.1	89.3 ± 2.9

^a^ The inhibition parameters were obtained following non-linear regression analysis of direct inhibition of FXIa in Tris-HCl buffer, pH 7.4, containing 0.02% Tween80, 0.1% PEG8000, and 150 mM NaCl at 37 °C. Inhibition values were obtained by the spectrophotometric measurement of residual FXIa activity. ^b^ Errors represent ± 1 S.E.

**Table 4:** Hydrolysis of the chromogenic substrate S-2366 by FXIa in the presence of LSAS ^a^.

[1] (µg/mL)	S-2366 K_M_ (mM)	V_MAX_ (mAU/min)
**0**	0.36 ± 0.03 ^b^	37.7 ± 1.0
**1**	0.29 ± 0.03	26.9 ± 0.7
**10**	0.31 ± 0.05	24.6 ± 1.2
**25**	0.31 ± 0.07	13.3 ± 0.9
**50**	0.43 ± 0.04	9.0 ± 0.3
**150**	0.30 ± 0.14	4.9 ± 0.7

^a^ K_M_ and V _MAX_ values of S-2366 chromogenic tripeptide substrate hydrolysis by human FXIa were spectrophotometrically determined by Michaelis–Menten kinetics. ^b^ Error represents ± 1 S.E.

**Table 5:** Inhibition profile of different polymers ^a^.

Polymer	FXIa IC_50_ (µg/mL)	Thrombin IC_50_ (µg/mL)	FXa IC_50_ (µg/mL)
**LSAS**	7.9 ± 0.9 ^b^	>125	>125
**LSAS acetate**	0.39 ± 0.1	0.73 ± 0.04	0.48 ± 0.09
**Desulfonated**	53.9 ± 16.3	*ND* ^c^	*ND*

^a^ The IC_50_ values were obtained following non-linear regression analysis of direct inhibition of human enzymes in Tris‒HCl buffers at 37 °C and pH 7.4. Inhibition was monitored by measuring the residual enzyme activity (UV or fluorescence). ^b^ Errors represent ± 1 S.E. ^c^ Not determined.

**Table 6:** Hydrolysis of the chromogenic substrate S-2366 by FXIa in the presence of desulfonated polymer ^a^.

Desulfonated Polymer (µg/mL)	S-2366 K_M_ (mM)	V_MAX_ (mAU/min)
**0**	0.25 ± 0.05 ^b^	32.1 ± 2.1
**10**	0.35 ± 0.04	29.7 ± 1.1
**50**	0.99 ± 0.24	17.8 ± 2.0
**250**	0.70 ± 0.12	10.6 ± 0.8

^a^ K_M_ and V _MAX_ values of S-2366 substrate hydrolysis by FXIa were measured by Michaelis–Menten kinetics. ^b^ Error represents ± 1 S.E.

## Data Availability

Data is contained within the article and Supplementary Material.

## Supplementary Materials

The following are available online at https://www.mdpi.com/article/10.3390/ph14090886/s1, Table S1. Effects of known molecular entities on APTT and PT in normal as well as deficient human plasmas, Figure S1: Absorbance of LSAS solution prepared in FXIa buffer (which contains 0.02% Tween80) showing no aggregation at the highest concentration tested,
