# Supplementary material for: Lignosulfonic Acid Sodium Is a Noncompetitive Inhibitor of Human Factor XIa
Source: Pharmaceuticals (Basel). 2021 Aug 31;14(9):886. doi: 10.3390/ph14090886 (PMC8466798; doi:10.3390/ph14090886)

Lignosulfonic Acid Sodium (LSAS) is Noncompetitive Inhibitor of Human Factor XIa

Srabani Kar,[1] Page Bankston,[1] Daniel K Afosah,[2] Rami A. Al-Horani[1]*

[1]*Division of Basic Pharmaceutical Sciences, College of Pharmacy, Xavier University of Louisiana,*
New Orleans LA 70125 USA

[2]*Department of Chemistry and Biochemistry, Washington and Lee University,*
Lexington VA 24450 USA

Table S1. Effects of known molecular entities on APTT and PT in normal as well as deficient human plasmas.

**Anticoagulants**	**APTT ($EC_{\times2}$)[a]**	**PT ($EC_{\times2}$)[a]**
LSAS (under investigation)	308.8 ± 23.9[b] µg/mL	980.1 ± 145.0
UFH (AT activator)	0.68 µg/mL<br>(AT Def. =10.1 µg/mL)	2.53 µg/mL
Argatroban HCl (Thrombin inhibitor)	0.29 ± 0.02 µM	0.34 ± 0.04 µM
Rivaroxaban (FXa inhibitor)	0.12 ± 0.003 µM	0.18 ± 0.05 µM
Anti-F11 (FXIa inhibitor)	1.99 µg/mL<br>(FXI Def. >>3.6 µg/mL)	>>3.6 µg/mL
C6B7 (FXIIa inhibitor)	~0.05 µg/mL<br>(FXII Def. >>0.06 µg/mL)	>>0.06 µg/mL

[a] The effective concentration to double the clotting time in the corresponding assay; [b] Error represents ± 1 SE. APTT: Activated partial thromboplastin time; PT: Prothrombin time; AT: Antithrombin.

Figure S1. Absorbance of LSAS solution prepared in FXIa buffer (which contains 0.02% Tween80) showing no aggregation at the highest concentration tested.

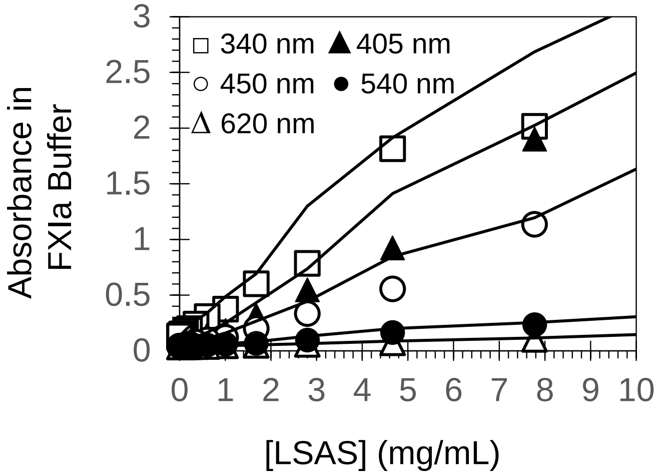

Supplement: Supplementary file 1 [file pharmaceuticals-14-00886-s001.zip › pharmaceuticals-1355499-supplementary.pdf]
